# Theranostic Imaging of Yttrium-90

**DOI:** 10.1155/2015/481279

**Published:** 2015-05-28

**Authors:** Chadwick L. Wright, Jun Zhang, Michael F. Tweedle, Michael V. Knopp, Nathan C. Hall

**Affiliations:** ^1^Department of Radiology, The Ohio State University Wexner Medical Center, Columbus, OH 43210, USA; ^2^Department of Radiology, Hospital of the University of Pennsylvania, Philadelphia, PA 19104, USA

## Abstract

This paper overviews Yttrium-90 (^90^Y) as a theranostic and nuclear medicine imaging of ^90^Y radioactivity with bremsstrahlung imaging and positron emission tomography. In addition, detection and optical imaging of ^90^Y radioactivity using Cerenkov luminescence will also be reviewed. Methods and approaches for qualitative and quantitative ^90^Y imaging will be briefly discussed. Although challenges remain for ^90^Y imaging, continued clinical demand for predictive imaging response assessment and target/nontarget dosimetry will drive research and technical innovation to provide greater clinical utility of ^90^Y as a theranostic agent.

## 1. Yttrium-90 and Its Role in Targeted Radiotherapy

In general, theranostics are agents that possess diagnostic and therapeutic attributes for personalized patient treatment for various diseases [1]. A commonly used theranostic agent is radioactive iodine (e.g., Iodine-131 or ^131^I) for the evaluation of thyroid physiology and pathophysiology, treatment of hyperthyroidism, treatment of thyroid cancer, and posttreatment assessment of radioactive iodine distribution in the body. The rare-earth lanthanide, Yttrium-90 (^90^Y), is almost exclusively a high-energy beta-particle (i.e., electron or *β* ^−^) emitting radionuclide used for radiotherapy with a maximum particle energy of 2.28 MeV (average energy of 0.94 MeV) that allows for high dose deposition with an average and maximum soft tissue penetration of 2.5 mm and 11 mm, respectively [2, 3]. ^90^Y has a physical half-life of 64.1 h [4] which makes it amenable for a variety of targeted radiotherapy applications including ^90^Y-labeled colloid [5, 6], somatostatin-receptor targeting peptides [7, 8], tumor-targeting antibodies [9, 10], and resin/glass microspheres for catheter-directed embolization of hepatic malignancy and metastases [3, 11–13]. Regardless of the targeted delivery agent used, the selection of ^90^Y and its use for radiotherapy are complex and necessitate close collaboration among various medical specialties including nuclear medicine, interventional radiology, medical oncology, and radiation medicine [14]. ^90^Y can be administered via direct injection into a space or cavity (e.g., radiosynovectomy), intravenously for peptide receptor radionuclide therapy (PRRT) and radioimmunotherapy (RIT), and intra-arterially for radioembolization (RE) therapy.

**Figure 1:**
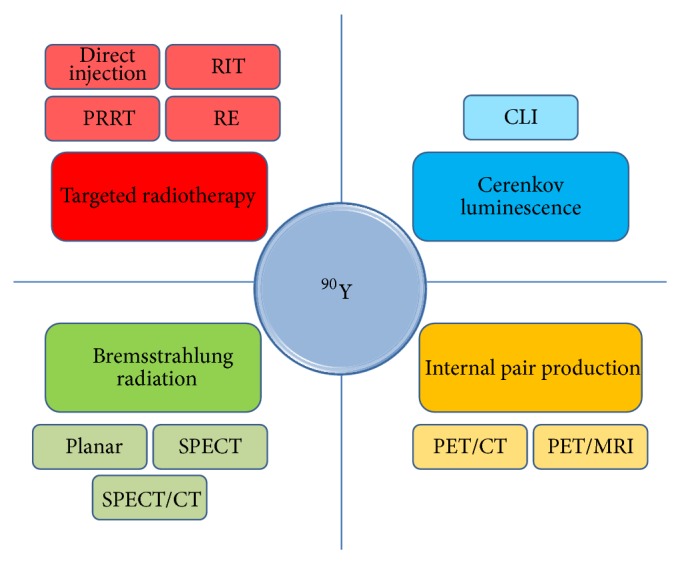
Yttrium-90 as a theranostic agent (i.e., it demonstrates both therapeutic and diagnostic attributes). Yttrium-90 (^90^Y, center) is a high-energy *β* ^−^ emitting radioisotope used clinically for targeted radiotherapy (upper left). The targeted radiotherapy applications include direct injection of ^90^Y into a body space or cavity, conjugation of ^90^Y to a peptide for peptide receptor radionuclide therapy (PRRT), or an antibody for radioimmunotherapy (RIT), or incorporation of ^90^Y into a resin or glass microsphere for radioembolization (RE) therapy. The high-energy *β* ^−^ particle emission produces a continuous spectrum bremsstrahlung radiation which can then be imaged using conventional nuclear medicine imaging systems such as planar gamma cameras, SPECT, and SPECT/CT (lower left). Although the vast majority of ^90^Y decays are *β* ^−^ emitting, 32 per million ^90^Y decays result in internal pair production that can be readily imaged using conventional PET/CT and PET/MRI systems (lower right). The high-energy *β* ^−^ particle emission also produces continuous spectrum light photons or Cerenkov luminescence which can then be imaged using existing bioluminescence imaging systems (upper right). These 3 noninvasive imaging approaches allow for simultaneous diagnostic assessment/localization of the therapeutic ^90^Y radioactivity.

Other therapeutic *β* ^−^ emitting radioisotopes (e.g., ^131^I for thyroid cancer [15] and Samarium-153 (^153^Sm) for osseous metastases [16]) also produce discrete gamma photons which can be imaged after therapy but contribute to additional absorbed radiation dose. One advantage of ^90^Y is that it is an almost pure *β* ^−^ emitting radioisotope which lacks such gamma photons [6]. On the other hand, because of the lack of gamma photons from ^90^Y, conventional scintigraphic imaging and assessment of the posttherapy distribution of its radioactivity are challenging. This lack of gamma photons led to the development and use of surrogate gamma-emitting radioisotopes (e.g., Indium-111- (^111^In-) labeled peptides and antibodies) with analogous chemical properties as a tracer for ^90^Y dosimetric assessment and pharmacokinetics [2, 17]. Likewise, Technetium-99m- (^99m^Tc-) labeled macroaggregated albumin (MAA) is currently used as a surrogate radiotracer for planning ^90^Y microsphere RE therapy [18–20]. It is important to note that use of such surrogate tracers may not always accurately predict ^90^Y radiotherapy effects* in vivo* and such discrepancies may result in unanticipated and unintended toxicities [17, 21–23]. Given that surrogate tracer agents may not always predict the precise posttherapeutic distribution of ^90^Y, subsequent imaging assessment of ^90^Y radioactivity is an important adjunctive step to assess and verify delivery and dosimetric distribution of the ^90^Y agent to the target(s) and exclude any nontargeted delivery [24]. Likewise, accurate quantification of ^90^Y radioactivity in both targeted lesions and nontargeted tissues would allow for improved comparisons of radiotherapy outcomes in patients. This review will subsequently discuss the different diagnostic imaging approaches used for therapeutic ^90^Y radioactivity assessment (Figure 1).

## 2. Bremsstrahlung Radiation

Conventional scintigraphic imaging and quantification of monoenergetic gamma-emitting medical radioisotopes (e.g., ^99m^Tc) have driven the evolution of current planar gamma cameras with optimized collimators and detector crystals for detecting and counting primary (i.e., unscattered) photons in discrete energy windows. *β* ^−^ particle emission from ^90^Y produces bremsstrahlung photons which can also be imaged scintigraphically [6, 25]. The ^90^Y bremsstrahlung photons are generated when the high-energy *β* ^−^ particle (i.e., electron) is emitted from the ^90^Y nucleus and then slows (i.e., it loses its kinetic energy) while interacting with adjacent atoms. As the electron slows down, its kinetic energy is converted into the continuous energy spectrum of both primary and scattered photons with no dominant energy photopeak for conventional scintigraphic imaging (i.e., bremsstrahlung radiation).

In 1967, Simon and Feitelberg described posttherapy bremsstrahlung imaging assessment of intra-arterially administered ^90^Y-labeled plastic microspheres in oncology patients [25]. Furthermore, they described an early clinical case of nontargeted deposition of ^90^Y-labeled microspheres within the lungs of a patient with a radioembolized left renal mass. The radioembolized left renal mass and bilateral lungs demonstrated ^90^Y radioactivity on posttherapy bremsstrahlung imaging and the bilateral lung radioactivity was presumed arteriovenous shunting of microspheres through the tumor and then trapped in the lungs. Subsequently, others have described posttherapy planar bremsstrahlung imaging for patients following direct injection of ^90^Y (e.g., radiosynovectomy) [5, 6], intravenous administration of ^90^Y-labeled RIT [26], and intra-arterial administration of ^90^Y-labeled microspheres [27–31]. In addition, one study demonstrated the capability for planar bremsstrahlung imaging to detect focal ^90^Y radioactivity using a phantom model simulating soft tissue extravasation of an intravenous ^90^Y dose [32].

Although technically feasible, image quality for ^90^Y bremsstrahlung is limited by overlying tissue attenuation, internal photon scattering, variable count rates of emitted bremsstrahlung photons, a wide range of photon energies produced, low spatial resolution (which worsens with increasing source distances to the camera), type of collimation employed (i.e., low, medium, or high-energy collimators), and image processing. In particular, attenuation coefficients may not be constant for the range of photon energies acquired by the gamma camera. Likewise, lower energy bremsstrahlung photons are more likely to scatter than high-energy photons. On the other hand, higher energy photons are more likely to penetrate collimator septae and detector crystals which degrade image quality and limit quantification [6, 33–37]. No standardized imaging protocol was used for these early ^90^Y bremsstrahlung imaging studies. Subsequent efforts to optimize planar ^90^Y bremsstrahlung imaging have used Monte Carlo simulation modeling [35] and these efforts support the use of medium or high-energy parallel-hole collimators and energy windows ranging from 50 to 200 keV. Quantification of ^90^Y bremsstrahlung radioactivity is likewise challenging but advances in both qualitative ^90^Y bremsstrahlung imaging and quantitative ^90^Y bremsstrahlung imaging have been described using optimized photon energy windows, collimation, attenuation correction, image filtering, and reconstruction [2, 24, 33, 34, 37–44].

It should be noted though that planar quantification is a two-dimensional (2D) assessment of ^90^Y radioactivity with limited potential for distinguishing overlapping sources of ^90^Y radioactivity [38]. Compared to planar imaging, the application of single photon emission computed tomography (SPECT) to ^90^Y bremsstrahlung imaging allows for improved three-dimensional (3D) visualization and anatomic discrimination of discrete adjacent foci of ^90^Y radioactivity as well as improving the potential for quantification [6]. The use of medium- and high-energy parallel-hole collimation is again supported to optimize camera sensitivity for ^90^Y bremsstrahlung photons but, like planar imaging, SPECT cannot distinguish between primary and scattered bremsstrahlung photons and this limits quantitation [2, 45]. The fusion of ^90^Y bremsstrahlung SPECT with X-ray computed tomography (CT) allows for attenuation correction and 3D anatomical localization of SPECT findings (i.e., SPECT/CT) [38]. This represents another distinct advantage over bremsstrahlung 2D planar and 3D SPECT only imaging [46].

In 1988, ^90^Y bremsstrahlung SPECT imaging was described in patients following direct injection of ^90^Y-colloid (i.e., radiosynovectomy) and confirmed ^90^Y bremsstrahlung radioactivity within the complex 3D knee joint space [6]. Subsequently several other clinical studies have described posttherapy SPECT and/or SPECT/CT bremsstrahlung imaging for patients following direct injection of ^90^Y [47], intravenous administration of ^90^Y-labeled RIT [2, 26] and PRRT [47], and intra-arterial administration of ^90^Y-labeled microspheres (resin [14, 29–31, 44, 48–59], glass [54, 60–63], or not specified [64]).  Table 1 lists the previously reported image acquisition settings used for clinical ^90^Y bremsstrahlung planar and SPECT imaging. The American Association of Physicists in Medicine (AAPM) has issued recommendations for post-RE bremsstrahlung imaging in 2011 which included the use of medium-energy collimation and an energy window of 68–92 keV [65].

Given that SPECT imaging requires much more time than planar imaging approaches, planar ^90^Y bremsstrahlung imaging can be more readily adopted for whole-body assessment of ^90^Y distribution [38]. On the other hand, bremsstrahlung SPECT imaging may allow for improved quantification when compared with planar approaches and better 3D dose assessment of localized ^90^Y radioactivity [36]. Recently, bremsstrahlung SPECT/CT imaging has been the imaging modality of choice for qualitative post-^90^Y RE assessment of liver radioactivity but image quality is still less than ideal [14, 65].

## 3. Internal Pair Production

Although the vast majority of ^90^Y decays result in therapeutic *β* ^−^ particle emission, 32 per million decays result in internal pair production that produces annihilation radiation that can be also imaged* in vitro* using positron emission tomography (PET) imaging systems [66–68]. While this rate of internal pair production is very small, there is a detectable peak of 511 keV photons which exceeds the continuous spectrum of bremsstrahlung photons and these 511 keV photons can be detected and imaged using conventional PET imaging [66]. PET detection of ^90^Y internal pair production represents a promising approach for even more accurate ^90^Y quantification* in vitro* and* in vivo* by minimizing the previously noted challenges associated with ^90^Y bremsstrahlung imaging [67].

These observations led to the first clinical case report, in 2009, of PET/CT imaging of ^90^Y radioactivity following ^90^Y-labeled resin microsphere RE for colorectal liver metastases, which demonstrated the feasibility of imaging ^90^Y* in vivo* using an existing conventional PET/CT system [50]. The detected intrahepatic ^90^Y radioactivity correlated well with the targeted intrahepatic lesion. Likewise, quantitative assessments of ^90^Y radioactivity in phantoms could also be performed with further improvement in quantitative accuracy using Time-of-Flight (ToF) PET reconstruction [44, 69, 70]. ToF PET imaging demonstrates some advantages in ^90^Y radioactivity assessment when compared with non-ToF PET imaging systems [71] and ^90^Y bremsstrahlung SPECT/CT imaging [40, 51]. Subsequently several other clinical studies have described posttherapy ^90^Y internal pair production PET imaging for patients following direct injection of ^90^Y [47], intravenous administration of ^90^Y-labeled RIT [54] and PRRT [47], and intra-arterial administration of ^90^Y-labeled microspheres (resin [14, 20, 28, 44, 51–55, 58, 70, 72–77], glass [54, 61, 62, 78], or not specified [64, 79]).

Image quality for ^90^Y internal pair production is limited by its very small branching fraction (i.e., 32 per million decays) and therefore necessitates longer acquisition times than traditional positron-emitting radioisotopes (e.g., Fluorine-18 (^18^F) which has a branching fraction of 967 per 1000 decays). It was also noted that measureable background radioactivity was dependent upon the PET imaging system used. The presence of a small fraction of radioactive Lutetium-176 (^176^Lu) within the detection crystals (i.e., lutetium yttrium orthosilicate or LYSO or lutetium oxyorthosilicate or LSO) of PET imaging systems contributes to this measureable background radioactivity [69, 78]. This requires that ^176^Lu background radioactivity be corrected for in order to obtain any accurate ^90^Y radioactivity assessment using these PET systems [78]. The ^176^Lu background radioactivity is not present on PET imaging systems which utilize bismuth germinate (BGO) detector crystals [66] and the BGO PET can provide ^90^Y radioactivity quantification [80]. It has been reported that BGO PET systems may be less accurate for ^90^Y radioactivity quantification when compared with LYSO-dependent PET systems due to the slower response rate and poorer contrast performance of BGO PET systems [71]. There are no reported clinical instances of PET detector saturation from ^90^Y bremsstrahlung radiation.

Despite the low branching fraction for ^90^Y and background radioactivity of some PET imaging systems, PET/CT imaging demonstrates better spatial resolution and image contrast than bremsstrahlung imaging (planar, SPECT, and SPECT/CT) [28, 44, 51] and clinically demonstrates improved detection of nontarget ^90^Y radioactivity compared with even bremsstrahlung SPECT/CT [14]. Although ^90^Y internal pair production imaging has been studied* in vitro* and* in vivo* using a variety of different PET imaging systems, different acquisition times, and different reconstruction algorithms, no standardized or consensus imaging protocol has been described for ^90^Y PET/CT imaging studies to date.  Table 2 details some of the acquisition and image reconstruction parameters used for clinical ^90^Y internal pair production PET imaging studies. In 2013, Kao et al. [14] described a diagnostic reporting approach for ^90^Y PET/CT imaging following RE therapy in order to (1) confirm successful deposition of the ^90^Y microspheres within the target lesion(s) and (2) detect any nontarget ^90^Y radioactivity. In this study, ^90^Y PET/CT imaging was consistently superior to ^90^Y bremsstrahlung SPECT/CT imaging in the qualitative assessment of post-RE patients, especially in the detection of nontarget ^90^Y radioactivity [58].

## 4. Cerenkov Luminescence

Another innovative approach for imaging of ^90^Y is real time detection of Cerenkov radiation (CR), that is, ultraviolet and visible light emitted in the presence of high-energy *β* ^−^ particle and positron-emitting radionuclides [81–83]. CR is produced when electrons or positrons travel faster than the speed of light through an aqueous medium (i.e., cells, tissues, and organs). As these high-energy charged particles travel through water, they disrupt the local electromagnetic field in the water. Electrons in the atoms of the water molecules will be displaced, and the atoms become polarized by the passing electromagnetic field of the *β* ^−^ particle or positron. Visible and ultraviolet light photons are emitted as the displaced electrons in the water molecules restore themselves to equilibrium and these light photons can be detected with existing high-sensitivity bioluminescence imaging systems. This optical imaging of CR has been designated as Cerenkov luminescence imaging (CLI) [84]. Detectable CLI signals have been described* in vitro* for a number of positron-emitting radioisotopes (e.g., ^18^F, Gallium-68, or ^68^Ga) and *β* ^−^ particle emitting radioisotope (e.g., ^90^Y and ^131^I) [85–87]. To date, ^90^Y is the most efficient medical radioisotope for Cerenkov luminescence production [85]. In preclinical studies,* in vivo* CLI has been performed in mouse models following intravenous administration of ^90^Y salt solution [85] and ^90^Y-labeled peptide [85, 88].

This novel optical imaging approach for noninvasively detecting ^90^Y radioactivity* in vitro* and* in vivo* presents many exciting opportunities. High spatial resolution images of ^90^Y radioactivity using CLI can be obtained within seconds as opposed to several minutes with conventional planar, SPECT, and PET imaging systems. CLI systems also allow for imaging multiple animals simultaneously as opposed to individually using micro-SPECT/PET imaging systems. These CLI systems are also much less expensive when compared with conventional- or micro-SPECT/PET imaging systems. This CLI approach for the preclinical development of targeted ^90^Y theranostics (e.g., nanoparticles, microspheres, colloids, peptides, and antibodies) will be tremendously enabled for researchers and clinicians. Clinical proof-of-concept (i.e., human Cerenkography) has recently been described for radiotherapy using ^131^I [89]. To date, no clinical applications for ^90^Y Cerenkography have been described in the literature.

## 5. Challenges and Future Directions for ^**90**^ **Y** Imaging

One current challenge for ^90^Y imaging is the lack of consensus guidelines for the technical acquisition, imaging reconstruction, and qualitative/quantitative interpretation of planar, SPECT, and PET imaging by the nuclear medicine community (e.g., Society of Nuclear Medicine and Molecular Imaging (SNMMI) and European Association of Nuclear Medicine (EANM)). An initial consensus guideline would establish the basis for future imaging studies to design, develop, and optimize ^90^Y imaging approaches and reporting. Likewise, a consensus guideline would describe relevant imaging signs following ^90^Y radiotherapy for imagers [63]. Another closely related challenge is that the vast majority of nuclear medicine imaging systems in place around the world are not currently designed or specifically optimized for ^90^Y imaging applications. While some manufacturers have provided assistance and expertise to adapt existing imaging systems for ^90^Y imaging [46], most imaging centers may have to internally customize imaging protocols with little guidance or validation. It is critical that professional organizations, nuclear medicine physicians, and researchers continue to interface and actively engage the imaging system manufacturers to develop and optimize specific protocols for more consistent and comparable ^90^Y image acquisition, image reconstruction, and, ideally, quantification. In addition, new technical advances incorporated into the state-of-the-art PET/CT imaging systems like digital PET/CT and continuous bed motion PET acquisition will need to be methodically assessed for advantages and limitations. Although a single case report on respiratory-gated PET/CT imaging for ^90^Y RE has been described [79], the advantages and limitations of respiratory-gated ^90^Y PET imaging will also need to be addressed.

**Figure 2:**
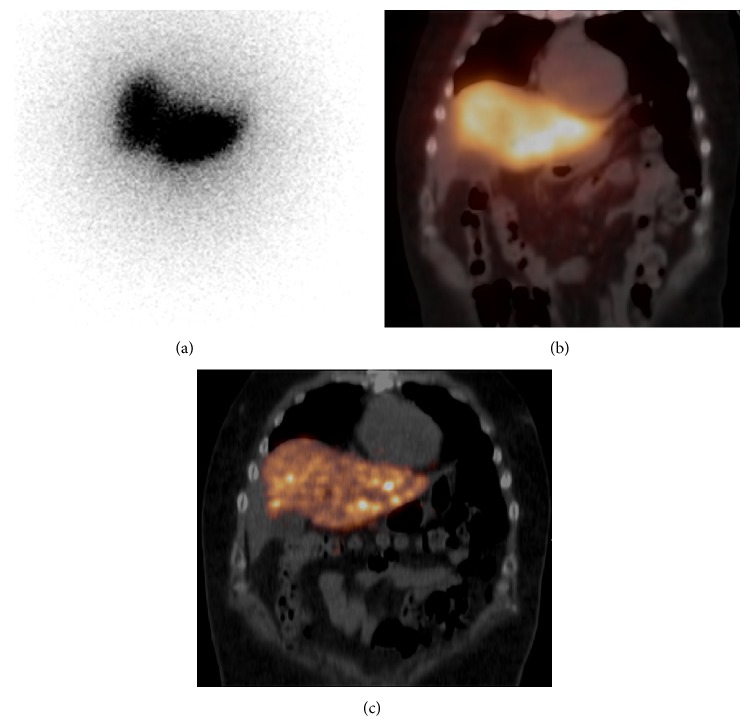
Imaging ^90^Y bremsstrahlung and internal pair production following ^90^Y microsphere RE therapy. This patient underwent intra-arterial administration of 1.74 GBq of ^90^Y-labeled glass microspheres to the left hepatic lobe for the treatment of colorectal metastases. Post-RE therapy imaging included ^90^Y bremsstrahlung planar and SPECT/CT imaging as well as ^90^Y internal pair production PET/CT imaging. Bremsstrahlung planar and SPECT/CT imaging was obtained using the Symbia T16 system with medium-energy collimation (Siemens Healthcare). Bremsstrahlung photons were imaged using an energy window of 111–150 keV and were reconstructed using FLASH3D (8 iterations, 4 subsets). Internal pair production PET/CT imaging was obtained with the Gemini 64 Time-of-Flight system (Philips Healthcare). PET data were reconstructed using a 3D line-of-response TOF blob-based algorithm (3 iterations, 33 subsets). (a) Two-dimensional planar bremsstrahlung image of the abdomen (anterior view) which demonstrates intense bremsstrahlung activity corresponding to left hepatic lobe region as well as the presence of scattered photons in the field of view emanating from the treated left hepatic lobe. (b) Three-dimensional bremsstrahlung SPECT/CT image of the abdomen (fused SPECT/CT in the coronal plane) again demonstrates bremsstrahlung activity corresponding to the left hepatic lobe. Like the planar image, the fused SPECT/CT image demonstrates the presence of additional scattered photons and this additional scatter activity overlies several adjacent soft tissues and organs (e.g., heart, chest wall, right hepatic lobe, gallbladder, and bowel). (c) Three-dimensional internal pair production PET/CT image of the abdomen (fused PET/CT in the coronal plane) demonstrates ^90^Y activity within the left hepatic lobe with more precise delineation of the ^90^Y activity within the liver and greatly improved ^90^Y-to-background contrast in the adjacent soft tissues and organs.

Recently, the trend in ^90^Y imaging has largely focused on 3D modalities like SPECT/CT and PET/CT (Figure 2). The majority of the literature relates to ^90^Y radioactivity imaging for post-RE assessment of ^90^Y-labeled resin microspheres using bremsstrahlung SPECT/CT and, more recently, internal pair production PET/CT. There are fewer reports related to the post-RE assessment of ^90^Y-labeled glass microspheres and even less related to ^90^Y imaging assessment of direct injection radiotherapies, RIT and PRRT. For the near future, ^90^Y internal pair production PET/CT will likely be compared with ^90^Y bremsstrahlung SPECT/CT imaging (i.e., a reference imaging standard). Although PET/CT imaging systems are more readily accessible today, ^90^Y PET imaging may be more challenging to incorporate into routine clinical workflows due to the low branching fraction and corresponding low count rates for ^90^Y (i.e., it requires longer acquisition times per bed position than more traditional ^18^F-fluorodeoxyglucose PET/CT imaging studies) [61]. There is a single case report for ^90^Y imaging with PET integrated with magnetic resonance imaging (MRI) [62]. Given that even fewer PET/MRI imaging systems are available than PET/CTs, it will be important that future studies address the advantages and limitations of PET/MRI imaging over PET/CT.

Review of current literature suggests that ^90^Y bremsstrahlung SPECT/CT imaging will continue in the future as (1) a reference standard for comparing different ^90^Y imaging modalities and (2) a more widely accessible imaging modality for qualitative assessment of ^90^Y radioactivity. As such, continued technical and methodological advances will likely improve SPECT/CT image quality, consistency, and quantification. Although ^90^Y bremsstrahlung imaging is better with SPECT/CT than planar imaging, planar imaging approaches may represent a more accessible and less expensive qualitative imaging modality capable of performing faster whole-body assessment of ^90^Y radioactivity than existing SPECT/CT technology. If any gross irregularity is detected with qualitative planar imaging, the patient could be referred for SPECT/CT or PET/CT assessment. The ever-present limitation of 2D planar bremsstrahlung imaging of ^90^Y radioactivity is the inability to resolve adjacent foci of ^90^Y radioactivity in target and nontarget tissues. In terms of patient safety and quality control/assurance during ^90^Y radiotherapy administration (e.g., direct cavity injection, intravenous and intra-arterial), planar bremsstrahlung imaging may play an important role in the future to document successful administration, confirm systemic circulation for nonembolic agents, and exclude any focal soft tissue extravasation or nontarget ^90^Y radioactivity. To this end, it has been recently proposed to optimize conventional Anger camera technology for interventional ^90^Y bremsstrahlung imaging applications [90].

Another exciting potential imaging modality for ^90^Y assessment is CLI. This technology may help to facilitate rapid and more cost-effective preclinical development of a wide array of targeted ^90^Y-labeled theranostic agents. One challenge for clinical implementation for CLI is the current requirement for no ambient light within the field of view of the CLI system (i.e., the sample, specimen, or subject must be imaged in total darkness). Ambient light can saturate the highly sensitive CLI imaging system and obscure the true Cerenkov luminescence emissions. Despite this limitation and challenge, human Cerenkography following ^131^I radiotherapy has already been described [89]. Future studies will also determine the feasibility and practicality of incorporating this optical imaging technology into qualitative clinical assessment of radiotherapy administration (i.e., during and after direct injection into a body cavity or space, intravenous or intra-arterial administration) as well as* in vivo*/*ex vivo* assessment of posttherapy ^90^Y-labeled target or nontarget lesions using CLI-capable endoscopes and specimen analyzers.

An international collaborative project (metrology for molecular radiotherapy or MetroMRT) has been initiated to address the known variability in absorbed dose for patients following radiotherapy, including ^90^Y [91]. Recently, an approach for developing a primary standard for ^90^Y-labeled resin microspheres was described [92]. This approach involves the complete dissolution of the ^90^Y-labeled resin microspheres within the source vial in order to obtain a more homogeneous ^90^Y activity distribution followed by primary measurement of the triple to double coincidence ratio (TDCR) of the sample using both Cerenkov and liquid scintillation detection techniques. The goals for the MetroMRT project as well as other future collaborations will be to develop and validate new approaches for accurately calibrating, assessing, quantifying, and verifying patient dosimetry related to targeted molecular radiotherapy. Such approaches that are ultimately traceable to a primary standard will enable more accurate individual patient dosimetry.

Recognizing and addressing the challenges for multimodality ^90^Y imaging will impact future prospective clinical trials which investigate the efficacy and safety of new ^90^Y theranostics. The long-term value for improved qualitative and quantitative ^90^Y imaging will be in confirming targeted delivery of the theranostic agent, evaluating nontarget radioactivity, estimating the absorbed dose to the target lesion(s) and nontarget tissue(s), evaluating and predicting treatment response, assessing the predictive power of existing non-^90^Y surrogate imaging agents, and promoting personalized medicine.

## 6. Conclusions

^90^Y is a theranostic agent which has been used clinically for direct radiation therapy, RIT, PRRT, and RE but it has been and remains a challenging radiotracer in terms of conventional nuclear medicine imaging approaches. The utilization of ^90^Y targeted radiotherapies is anticipated to increase. There is continued interest in developing and validating noninvasive imaging strategies to assess both targeted ^90^Y radioactivity and nontargeted ^90^Y radioactivity that are readily accessible, easy to implement, easy to interpret, and reported in a concise and consistent manner. In general, the ^90^Y imaging approaches discussed in this review are compatible with a theranostic paradigm [93]. Intraprocedural and postprocedural imaging can assess the adequacy of targeted ^90^Y delivery and provide absorbed dose estimates for the target(s) and nontarget tissues. These novel imaging approaches have the potential to further improve the efficacy of targeted ^90^Y radiotherapies, provide objective treatment monitoring and assessment, and ensure patient safety. Further innovations in qualitative and quantitative nuclear medicine imaging of ^90^Y radioactivity will continue to impact posttherapy patient management in this era of personalized medicine. The potential for optical imaging of ^90^Y radioactivity* in vitro* and* in vivo* (and potentially* ex vivo*) using Cerenkov luminescence may promote more timely and cost-effective preclinical development of targeted theranostics. Clinical and interventional applications for ^90^Y CLI are also likely to evolve.

## Figures and Tables

**Table 1:** Image acquisition parameters used for clinical ^90^Y bremsstrahlung planar and SPECT imaging studies.

Reference	Imaging	^90^Y agent	Collimator	Energy window(s) keV	Attenuation correction
Smith et al. [6]	Planar	Silicate	Medium energy	60–200	No
Tehranipour et al. [27]	Planar	Resin microspheres	Medium energy	72–119	No
Minarik et al. [26]	Planar	Anti-CD20 antibody	High energy	105–195	No
Ahmadzadehfar et al. [31]	Planar	Resin microspheres	Medium energy	55–250	No
Ahmadzadehfar et al. [30]	Planar	Resin microspheres	Medium energy	55–250	No
Smith et al. [6]	SPECT	Silicate	Medium energy	60–200	No
Mansberg et al. [48]	SPECT	Resin microspheres	Medium energy	77–104	Yes
Flamen et al. [49]	SPECT	Resin microspheres	Medium energy	53–88 and 97–287	Yes
Minarik et al. [2]	SPECT	Anti-CD20 antibody	High energy	105–195	Yes
Lhommel et al. [50]	SPECT	Resin microspheres	Medium energy	77–104	Yes
Minarik et al. [26]	SPECT	Anti-CD20 antibody	High energy	105–195	Yes
Strigari et al. [29]	SPECT	Resin microspheres	Medium energy	55–245	Yes
Ahmadzadehfar et al. [31]	SPECT	Resin microspheres	Medium energy	55–250	Yes
Ahmadzadehfar et al. [30]	SPECT	Resin microspheres	Medium energy	55–250	Yes
Wissmeyer et al. [62]	SPECT	Glass microspheres	Medium energy	77–104	Yes
Fabbri et al. [47]	SPECT	DOTATOC	Medium energy	58–102 and 153–187	Yes
Elschot et al. [55]	SPECT	Resin microspheres	High energy	50–250	Yes
Elschot et al. [45]	SPECT	Resin microspheres	High energy	105–195	Yes
Kao et al. [14, 58]	SPECT	Resin microspheres	Medium energy	74–86	Yes
Padia et al. [61]	SPECT	Glass microspheres	Medium energy	57–100	Yes
Ulrich et al. [56]	SPECT	Resin microspheres	Medium energy	68–83	Yes
Wondergem et al. [57]	SPECT	Resin microspheres	High energy	50–250	Yes
Eaton et al. [59]	SPECT	Resin microspheres	Medium energy	55–95	Yes

**Table 2:** Acquisition and image reconstruction parameters used for clinical ^90^Y internal pair production PET imaging studies. *∗* indicates that the scanner was a hybrid PET/MRI system whereas all other scanners listed were PET/CT systems.

Reference	^90^Y agent	Scanner/manufacturer	Detector crystal	Non-ToF versus ToF	Image reconstruction (number of iterations and subsets used)
Lhommel et al. [50]	Resin microspheres	Gemini Philips	LYSO	ToF	8 iterations, 3 subsets

Lhommel et al. [69]	Resin microspheres	Gemini Philips	LYSO	ToF	2 iterations, 33 subsets

Werner et al. [28]	Resin microspheres	Biograph Hi-Rez 16 Siemens	LSO	Non-ToF	8 iterations, 16 subsets and 4 iterations, 8 subsets

Gates et al. [78]	Glass microspheres	Biograph 40 Siemens	LSO	Non-ToF	3 iteration, 21 subsets

Wissmeyer et al. [62]	Glass microspheres	Gemini PET/MRI^*∗*^ Philips	LYSO	ToF	3 iterations, 33 subsets

Bagni et al. [72]	Resin microspheres	Discovery ST GE	BGO	Non-ToF	2 iterations, 15 subsets

Fabbri et al. [47]	DOTATOC	ECAT-EXACT47 Siemens	BGO	Non-ToF	2 iterations, 4 subsets

Kao et al. [53]	Resin microspheres	Biograph WO Siemens	LSO	Non-ToF	2 iterations, 8 subsets

Carlier et al. [54]	Resin and glass microspheres and anti-CD20 antibody	Biograph mCT 40 Siemens	LSO	ToF and non-ToF	1 or 3 iterations, 21 or 24 subsets

Chang et al. [74]	Resin microspheres	Biograph mCT Siemens	LSO	ToF	3 iteration, 12 subsets

Elschot et al. [55]	Resin microspheres	Biograph mCT Siemens	LSO	ToF	3 iterations, 21 subsets

Elschot et al. [45]	Resin microspheres	Biograph mCT Siemens	LSO	ToF	3 iterations, 21 or 24 subsets

Kao et al. [14, 58]	Resin microspheres	Discovery 690 GE	LYSO	ToF	3 iterations, 18 subsets

Mamawan et al. [79]	Resin or glass microspheres	Biograph mCT 40 Siemens	LSO	ToF	2 iterations, 21 subsets

Bourgeois et al. [76]	Resin microspheres	Biograph mCT Siemens	LSO	ToF	1 iteration, 21 subsets

## Abbreviations

AAPM: American Association of Physicists in Medicine
BGO: Bismuth germinate
CLI: Cerenkov luminescence imaging
CR: Cerenkov radiation
CT: Computed tomography
EANM: European Association of Nuclear Medicine
FDG: Fluorodeoxyglucose
LSO: Lutetium oxyorthosilicate
LYSO: Lutetium yttrium orthosilicate
MAA: Macroaggregated albumin
MetroMRT: Metrology for molecular radiotherapy
MRI: Magnetic resonance imaging
PET: Positron emission tomography
PRRT: Peptide receptor radionuclide therapy
RE: Radioembolization
RIT: Radioimmunotherapy
SNMMI: Society of Nuclear Medicine and Molecular Imaging
SPECT: Single photon emission computed tomography
TDCR: Triple to double coincidence ratio
ToF: Time-of-Flight
2D: Two-dimensional
3D: Three-dimensional
^18^F: Fluorine-18
^68^Ga: Gallium-68
^90^Y: Yttrium-90
^99m^Tc: Technetium-99m
^111^In: Indium-111
^131^I: Iodine-131
^153^Sm: Samarium-153
^176^Lu: Lutetium-176.
